# Irsogladine maleate, a gastric mucosal protectant, suppresses intestinal polyp development in *Apc*-mutant mice

**DOI:** 10.18632/oncotarget.7082

**Published:** 2016-01-30

**Authors:** Wakana Onuma, Susumu Tomono, Shinngo Miyamoto, Gen Fujii, Takahiro Hamoya, Kyoko Fujimoto, Noriyuki Miyoshi, Fumio Fukai, Keiji Wakabayashi, Michihiro Mutoh

**Affiliations:** ^1^ Epidemiology and Prevention Division, Research Center for Cancer Prevention and Screening, National Cancer Center, Tokyo, Japan; ^2^ Faculty of Pharmaceutical Sciences, Tokyo University of Science, Chiba, Japan; ^3^ Graduate Division of Nutritional and Environmental Sciences, University of Shizuoka, Shizuoka, Japan; ^4^ Division of Carcinogenesis and Cancer Prevention, National Cancer Center Research Institute, Tokyo, Japan; ^5^ Division of Molecular Biology, Nagasaki International University, Nagasaki, Japan

**Keywords:** *Apc*-mutant mice, irsogladine maleate, NF-κB, reactive carbonyl species, cancer chemoprevention

## Abstract

This study aimed to identify gastric mucosal protectants that suppress intestinal tumorigenesis in a mouse model. We chose six gastric mucosal protectants (ecabet sodium hydrate, irsogladine maleate, rebamipide, sofalcone, teprenone and troxipide) and examined their effects on the activity of oxidative stress-related transcriptional factors, including AP-1, NF-jB, NRF2, p53 and STAT3, in Caco-2 cells using a luciferase reporter gene assay. Among the six protectants, irsogladine maleate clearly inhibited NF-jB and AP-1 transcriptional activity. Furthermore, the chemopreventive property of irsogladine maleate was examined in a Min mouse model of familial adenomatous polyposis. Treatment with irsogladine maleate at doses of 5 and 50 ppm significantly reduced the number of intestinal polyps to 69% and 66% of the untreated control value, respectively. In these polyps, mRNA levels of the downstream targets of NF-jB, such as IL-1β and IL-6, were decreased by irsogladine maleate treatment. Moreover, the levels of oxidative stress-related markers, reactive carbonyl species, in the livers of Min mice were clearly decreased following the administration of irsogladine maleate. This study demonstrated that irsogladine maleate suppresses intestinal polyp formation in Min mice partly through the NF-jB signaling pathway, thus reducing oxidative stress.

## INTRODUCTION

It is important to establish effective preventive methods against colorectal cancer (CRC) as it currently accounts for approximately 8% of all cancer deaths worldwide [1] and is the fourth leading cause of cancer deaths. One potentially effective prevention strategy is use of aspirin, a classical non-steroidal anti-inflammatory drug (NSAID) [2–4]. Recently, we investigated the effects of low-dose, enteric-coated aspirin tablets in a double-blind, randomized, placebo-controlled clinical trial [5]. The participants were patients whose single/multiple colorectal tumors had all been excised by endoscopy. We successfully demonstrated for the first time that low-dose, enteric-coated aspirin therapy suppresses colorectal tumor recurrence in Asian patients (OR = 0.60, 95% CI: 0.36–0.98), and the suppressive effects were more significant in non-smokers than smokers. These results are in line with observations obtained in other colorectal tumor recurrence trials in Western countries using aspirin [6].

However, in addition to its valuable effects, aspirin has adverse effects, such as gastrointestinal bleeding and hemorrhagic stroke [7]. As aspirin is a nonselective and irreversible cyclooxygenase (COX)-inhibitor, aspirin inhibits platelet aggregation and can reduce thrombosis. To overcome these adverse effects, several approaches can be considered: (i) a reduction of the doses and treatment periods, (ii) the co-prescription of a gastric mucosal protectant, (iii) the eradication of *Helicobacter pylori* infection, which is a risk for gastrointestinal bleeding and (iv) the establishment of age restriction for aspirin use, which may overcome bleeding complications. We hypothesized that combining aspirin administration with a gastric mucosal protectant would reduce the risk of gastrointestinal bleeding. In this study, we only aimed to identify gastric mucosal protectants that suppress intestinal tumorigenesis in a mouse model, and left for future experiments whether the combined treatment of aspirin and gastric mucosal protectants inhibits intestinal carcinogenesis while suppressing the side-effects of aspirin.

Well-known oxidative stress-related transcription factors, such as activator protein-1 (AP-1), nuclear factor-κB (NF-κB), NF-E2-related factor 2 (NRF2), p53 and signal transducer and activator of transcription 3 (STAT3) [8], have been implicated in carcinogenesis. Among these, NRF2 and p53 possess anti-carcinogenic potential when activated.

Through their anti-oxidative stress potential, gastric mucosal protectants may play a suppressive role on intestinal tumorigenesis. However, this potential has not been clarified, and screening methods that completely evaluate the activities of oxidative stress-related transcriptional factors have not been performed. Thus, we aimed to identify an ideal gastric mucosal protectant with anti-oxidative stress and anti-intestinal tumorigenesis potential. We chose six gastric mucosal protectants that have been clinically used: ecabet sodium hydrate, irsogladine maleate, rebamipide, sofalcone, teprenone and troxipide. These test reagents are not H2 blockers or proton pump inhibitors because strong inhibition of gastric acid is reported to induce hypergastrinemia, which enhances the growth of tumors in *Apc*-mutant Min mice [9]. In this study, we demonstrated that irsogladine maleate significantly suppressed NF-κB and AP-1 transcriptional activities and decreased intestinal tumorigenesis in Min mice. Based on the reduced levels of reactive carbonyl species (RCs) in Min mice treated with irsogladine maleate, the mechanisms underlying the suppressive effect of the drug on intestinal tumorigenesis are also discussed.

## RESULTS

### The effects of six gastric mucosal protectants on AP-1, NF-κB, NRF2, p53 and STAT3 transcriptional activity in Caco-2 cells

**Figure 1:**
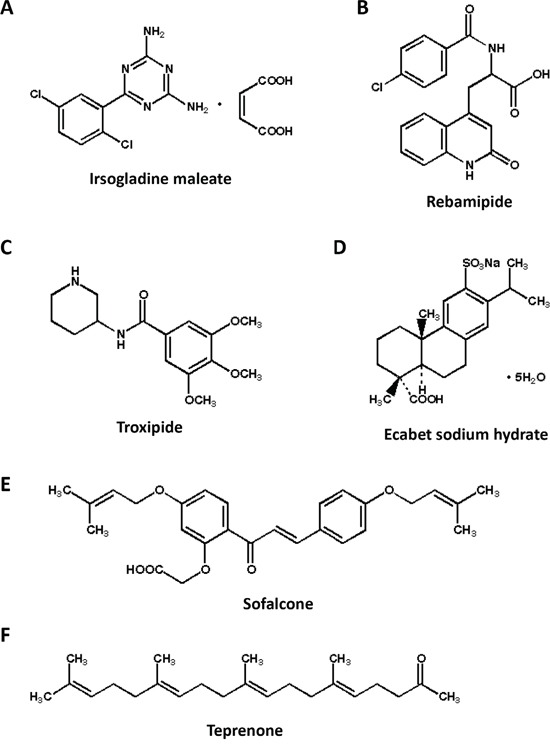
Chemical structures of six gastric mucosal protectants Irsogladine maleate **A.** rebamipide **B.** troxipide **C.** ecabet sodium hydrate **D.** sofalcon **E.** and teprenone **F**.

**Figure 2:**
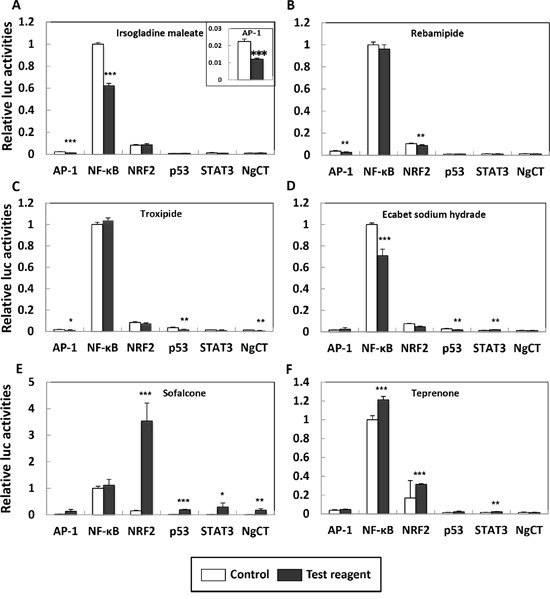
Effects of six gastric mucosal protectants on AP-1, NF-κB, NRF2, p53 and STAT3 transcriptional activation in Caco-2 cells Irsogladine maleate **A.** rebamipide **B.** troxipide **C.** ecabet sodium hydrate **D.** sofalcone **E.** and teprenone **F.** After transient transfection of the indicated reporter plasmid for 24 h, Caco-2 cells were treated with 200 μM of the test reagent for 24 h (closed box), or for 24 h without the test reagent (open box). The basal luciferase activity of NF-κB in the control was set as 1.0. A larger magnification of AP-1 transcriptional activity in Figure 2A is inserted within the square. The data are the means ± SD (n = 3), ***p < 0.001, **p < 0.01, *p < 0.05 *vs* control. NgCT: negative control.

AP-1, NF-κB, NRF2, p53 and STAT3 transcriptional activities were tested with six test reagents at a dose of 200 μM each for 24 hours (Figure 1 and 2). Irsogladine maleate decreased AP-1 and NF-κB transcriptional activities by 47% and 38% of the untreated control value, respectively (Figure 2A). Rebamipide decreased NRF2 transcriptional activities by 16% of the untreated control value, although it decreased AP-1 by 30% (Figure 2B). Ecabet sodium hydrate decreased NF-κB and p53 transcriptional activities by 29% and 36% of the untreated control value, respectively, but increased STAT3 transcriptional activity by 61% (Figure 2D). Teprenone increased NF-κB, increased NRF2 and decreased STAT3 transcriptional activities (Figure 2F). Troxipide and sofalcone had disturbance effects on the negative control, and their data were not included in the analysis (Figure 2C and 2E). Among the test reagents, the data from irsogladine maleate were best aligned with the purpose of this study. As AP-1 and NF-κB play important roles in the early stage of intestinal tumorigenesis, we selected irsogladine maleate for further *in vivo* experiments.

### The suppression of NF-κB transcriptional activity by irsogladine maleate and NF-κB inhibitor

NF-κB transcriptional activity was examined following treatment with irsogladine maleate (100 and 200 μM) and an NF-κB inhibitor (10 μM SM-7368) in Caco-2 and HCT-15 cells (Figure S1). In Caco-2 cells, irsogladine maleate at doses of 100 and 200 μM for 24 hours treatment decreased NF-κB transcriptional activity by 16% and 31% of the untreated control value, respectively (Figure S1A). Similarly, in HCT-15 cells, irsogladine maleate at doses of 100 and 200 μM for 24 hours treatment decreased NF-κB transcriptional activity by 22% and 29% of the untreated control value, respectively (Figure S1D). SM-7368 at a dose of 10 μM for 24 hours treatment decreased NF-κB transcriptional activity by 70.5% of the untreated control value in Caco-2 cells and by 64.4% in HCT-15 cells.

Moreover, cytokine mixture (TNFα, IL-1β and EGF)-stimulated NF-κB transcriptional activity was examined following treatment with irsogladine maleate (100 and 200 μM) SM-7368 in Caco-2 cells for 24 and 48 hours (Figure S1B and C). A 24-h irsogladine maleate treatment at doses of 100 and 200 μM decreased cytokine mixture-stimulated NF-κB transcriptional activity by 8.3% and 11.3% of the untreated control value, respectively. A 48-h irsogladine maleate treatment at doses of 100 and 200 μM decreased NF-κB transcriptional activity by 18.9% (**p < 0.01 *vs* control) and 30.8% (***p < 0.001 *vs* control) of the untreated control value, respectively. Similarly, a 48-h irsogladine maleate treatment at doses of 100 and 200 μM decreased cytokine mixture-stimulated NF-κB transcriptional activity by 6.8% and 22.0% (***p < 0.001 *vs* control) of the untreated control value, respectively.

### The suppression of intestinal polyp formation in Min mice by irsogladine maleate

The administration of 5 and 50 ppm irsogladine maleate to Min mice for 8 weeks did not affect body weight, food intake or clinical signs throughout the experimental period. Average daily food intake did not differ significantly among the groups. In addition, there were no observed changes in organ weights that might have been attributable to toxicity. Of note, no clear histopathological changes were observed by irsogladine maleate treatment to the stomach (Figure S2). However, serum triglyceride levels were changed, and decreased with the administration of 5 and 50 ppm irsogladine maleate to 42.1% (*p* < 0.01 *vs* 0 ppm) and 73.4% of the untreated control value, respectively. In the serum free fatty acid and total cholesterol levels, no remarkable change was observed (Figure S3)

Table 1 summarizes the data for the number and distribution of intestinal polyps in the basal diet and irsogladine maleate-treated groups. Almost all polyps developed in the small intestine, with only a few developing in the colon. The representative photos of small intestinal polyps in the three groups are shown in Figure S4. The total number of polyps decreased with the administration of 5 and 50 ppm irsogladine maleate to 69.3% and 66.1% of the untreated control value, respectively. Polyp formation was reduced in the proximal segment by 61.5% (*p* < 0.01 *vs* basal diet) and in the distal segment by 21.9% (*p* < 0.05 *vs* basal diet) with the administration of 5 ppm irsogladine maleate; with the administration of 50 ppm irsogladine maleate, polyp formation in the proximal segment was reduced by 53.8% (*p* < 0.01 *vs* basal diet) and that in the middle segment was reduced by 39.7% (*p* < 0.01 *vs* basal diet). In the colon, no remarkable change was observed following treatment with irsogladine maleate.

**Table 1:** The number of intestinal polyps/mouse in Min mice treated with irsogladine maleate

Irsogladine maleate (ppm)		No. of polyps/mouse				
	No. of mice	Small intestine			Colon	Total
Proximal	Middle	Distal				
0	10	9.1 ± 3.2	14.1 ± 3.8	27.4 ± 4.8	0.5 ± 0.7	51.1 ± 8.1
5	10	3.5 ± 1.9**	10.2 ± 4.2	21.4 ± 5.3*	0.3 ± 0.5	35.4 ± 9.3**
50	10	4.2 ± 1.9**	8.5 ± 2.4**	20.6 ± 6.7	0.5 ± 0.7	33.8 ± 8.3*

Data are presented as the means ± SD.

Significantly different from the untreated control group at

* *p* < 0.05,

** *p* < 0.01.

Figure S5 shows the size distributions of the intestinal polyps in the basal diet and irsogladine maleate-treated groups. The maximal number of polyps was observed in the size range up to 3.0 mm in diameter. The administration of 5 ppm irsogladine maleate significantly reduced the numbers of polyps sized < 0.5 mm (*p* < 0.01 *vs* basal diet) and between 1.5 and 2.0 mm (*p* < 0.05 *vs* basal diet) in diameter. Fifty ppm irsogladine maleate also decreased the number of polyps sized < 0.5 mm (*p* < 0.01 *vs* basal diet) and those between 0.5 and 1.0 mm (*p* < 0.05 *vs* basal diet) in diameter.

To evaluate the suppressive effects of irsogladine maleate on intestinal polyp development in Min mice, proliferating cell nuclear antigen (PCNA) was measured in the cell nuclei by immunohistochemistry. The percentage of PCNA-positive cells in each polyp was slightly, but not significantly, reduced by irsogladine maleate treatment from 51.2% (0 ppm) to 44.3% (5 ppm) and 38.6% (50 ppm),.

### Suppression of the down-stream target genes of NF-κB in the intestinal polyps of Min mice by irsogladine maleate

**Figure 3:**
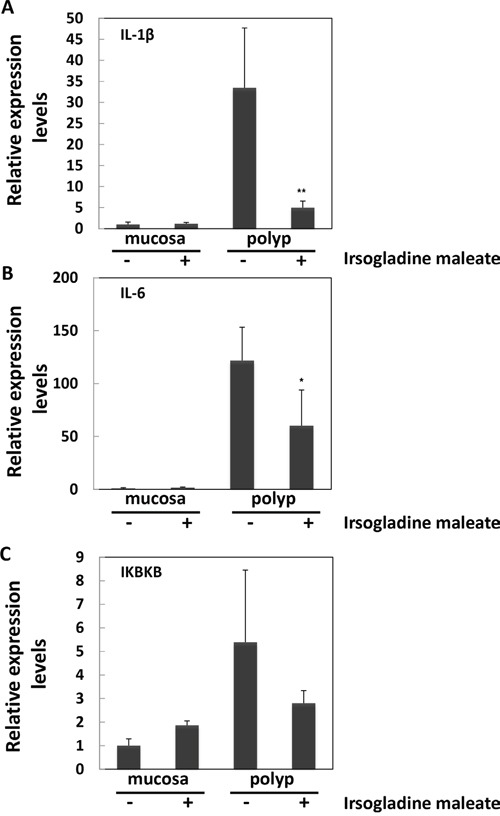
Suppression of the down-stream target genes of NF-κB in non-polyp intestinal mucosa segments and/or polyp segments of Min mice with or without 5 ppm irsogladine maleate treatment Quantitative real-time PCR analyses were performed to determine the IL-1β **A.** IL-6 **B.** and IKBKB **C.** mRNA expression levels in the polyps or non-polyp intestinal mucosa of Min mice who received diets containing irsogladine maleate at doses of 5 ppm for 8 weeks. The data are normalized according to GAPDH and presented as the means ± SD. **p < 0.01, *p < 0.05 *vs* 0 ppm.

The down-stream target genes of NF-κB in the intestinal polyp and non-polyp portions of Min mice were investigated. Real-time PCR revealed that treatment with 5 ppm irsogladine maleate for 8 weeks significantly suppressed interleukin (IL)-1β and IL-6 mRNA levels in the intestinal polyp segments by 85% and 51% of the untreated values, respectively (Figure 3). Inhibitor of nuclear factor kappa-B kinase subunit beta (IKBKB) mRNA levels tended to be reduced in intestinal polyp segments by 48% of the untreated value. The effects of 50 ppm irsogladine maleate on IL-1β and IL-6 mRNA levels were not significant (Figure S6).

### Reduction of the levels of oxidative stress-related markers and reactive carbonyl species in Min mice treated with irsogladine maleate

**Figure 4:**
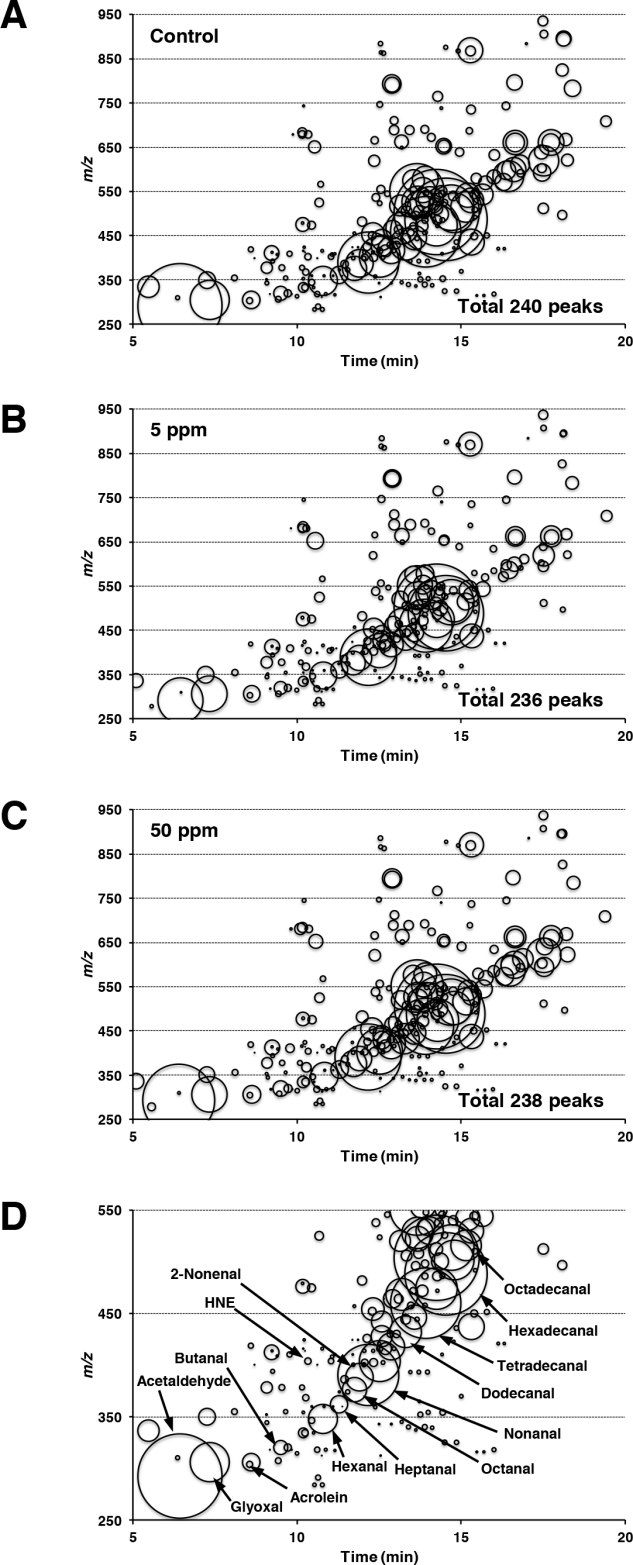
Corresponding RCs maps plotting free RCs detected in the liver samples All of the free RCs detected in the liver samples taken from 0 **A.** 5 **B.** or 50 **C.** ppm irsogladine maleate-treated Min mice are shown. RCs are plotted as circles as a function of their retention times (horizontal axis) and m/z values (vertical axis). The areas of the circles represent the intensities of the peaks of the detected RCs relative to that of the IS. Figure 4D is an enlarged view of Figure 4A, showing the m/z values in the range of 250 to 550, together with the names of certain RCs identified by the analyses. The RCs abbreviations are listed in the Materials and Methods section.

**Figure 5:**
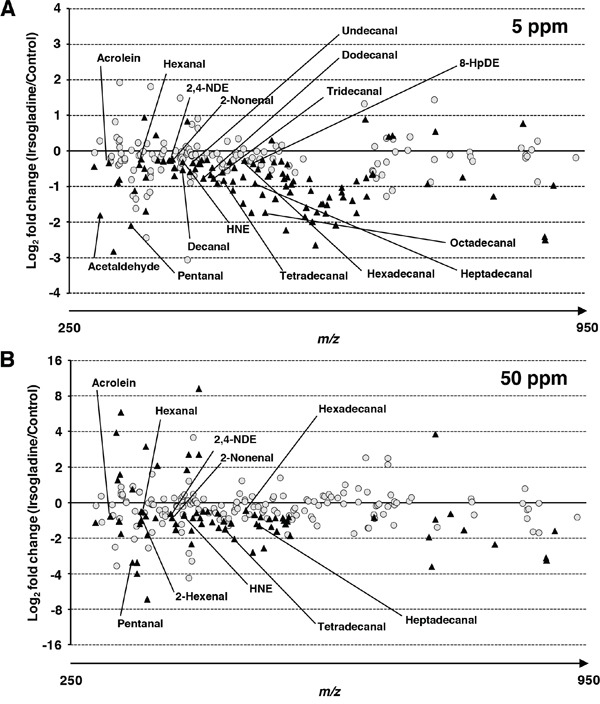
Relative RCs levels detected in liver samples taken from irsogladine maleate-treated mice compared with those of the non-treated mice The comparative RCs profiles of liver samples from 5 **A.** and 50 **B.** ppm irsogladine maleate-treated Min mice. The closed triangles indicate that the levels of the RCs were significantly different between the non-treated and irsogladine maleate-treated mice (*p* < 0.05).

Irsogladine maleate protects against intestinal polyp development in Min mice, potentially through the suppression of NF-κB-related oxidative stress. To examine this mechanism, we used liquid chromatography mass spectrometry (LC/MS) and detected the reactive carbonyl species in the liver of Min mice with or without irsogladine maleate treatment. LC/MS detected a total of 240, 236 and 238 peaks in the liver samples taken from the non-treated and the 5- and 50-ppm irsogladine maleate-treated Min mice, respectively. Of the 236 and 238 peaks detected, the levels of 194 and 163 peaks were lower in the 5- and 50-ppm irsogladine maleate-treated mice than in the non-treated mice, respectively, with the levels of 97 and 66 peaks being significantly lower (*p* < 0.05) (Figure 4). Through a comparison with authentic RCs, certain peaks were attributed to 32 RCs, including acrolein, pentanal, hexanal, 2,4-NDE, 2-nonenal, HNE, tetradecanal, hexadecanal and heptadecanal (Figure 5, Table 2). Table 2 presents the detected levels of these identified RCs in the liver samples of the irsogladine maleate-treated or non-treated mice. It is noteworthy that the levels of 17 identified RCs including typical DNA and/or protein damaging RCs were significantly lower in the liver samples taken from irsogladine maleate-treated mice than those of the non-treated mice.

**Table 2:** The levels of RCs detected in liver samples of irsogladine maleate-treated or non-treated Min mice

Compounds	Irsogladine maleate (ppm)		
	0	5	50
	pmol/g wet tissue		
Acetaldehyde	14242.2 ± 8478.3	4051.3 ± 4231.2**	9757.4 ± 8245.2
Acrolein	1239.5 ± 144.6	968.4 ± 303.1**	932.3 ± 211.9**
Glyoxal	8440.8 ± 3058.1	9864.9 ± 3368.4	6465.5 ± 3025.6
Propanal	1509.7 ± 534.4	1341.8 ± 369.4	1454.4 ± 431.8
Crotonaldehyde	5.4 ± 9.9	15.2 ± 19.8	22.8 ± 18.7*
Butanal	1205.2 ± 589.2	998.7 ± 356.6	1377.9 ± 409.7
Pentanal	31.4 ± 18.9	17.6 ± 12.3*	12.8 ± 17.4**
2-Hexenal	73.7 ± 14.2	68.1 ± 15.0	61.5 ± 9.9**
Hexanal	4388.3 ± 735.4	3726.9 ± 593.2*	3665.3 ± 531.9*
2-Heptenal	5.3 ± 9.1	3.1 ± 7.1	0.6 ± 1.3
Heptanal	651.2 ± 117.9	612.2 ± 89.6	627.5 ± 94.0
2-Octenal	14.7 ± 4.3	13.7 ± 3.3	12.6 ± 2.3
Octanal	1956.9 ± 466.0	1858.5 ± 292.3	2111.7 ± 335.1
2,4-NDE	147.9 ± 22.9	124.3 ± 17.6*	120.0 ± 15.3**
2-Nonenal	685.2 ± 100.7	565.5 ± 42.9**	502.9 ± 44.4***
Nonanal	5656.4 ± 2082.8	4800.1 ± 989.3	6214.0 ± 1546.3
2,4-DDE	53.5 ± 38.2	46.5 ± 36.8	35.0 ± 22.6
HNE	230.0 ± 34.2	186.7 ± 31.4**	181.9 ± 27.0**
Decanal	1728.3 ± 750.0	1192.5 ± 600.1*	2027.2 ± 269.8
EDE	174.3 ± 159.8	178.4 ± 131.0	207.7 ± 174.2
2-Undecenal	47.0 ± 8.1	43.7 ± 7.1	47.0 ± 8.3
Undecanal	761.0 ± 190.4	599.8 ± 154.7*	790.9 ± 119.4
Dodecanal	1337.7 ± 355.8	769.6 ± 456.9**	1147.1 ± 336.7
Tridecanal	1163.6 ± 330.2	673.8 ± 372.6**	952.4 ± 323.1
Tetradecanal	1250.9 ± 521.3	699.6 ± 154.0**	740.9 ± 207.6*
Pentadecanal	3437.1 ± 1311.8	2469.2 ± 857.4	3276.6 ± 1056.0
Hexadecanal	4141.7 ± 737.2	3442.5 ± 535.6*	3533.3 ± 595.1*
8,11,14-HpDTE	145.2 ± 69.2	123.0 ± 79.2	160.0 ± 124.7
8,11-HpDDE	113.0 ± 34.3	98.9 ± 24.5	104.1 ± 42.7
8-HpDE	215.8 ± 59.9	163.5 ± 31.8*	166.9 ± 60.9
Heptadecanal	877.7 ± 406.6	460.9 ± 129.2**	573.6 ± 207.2*
Octadecanal	616.5 ± 335.1	189.8 ± 157.0**	455.7 ± 226.7

Data are presented as the means ± SD (n = 10).

Significantly different from the untreated control group at

* *p* < 0.05

** *p* < 0.01

*** *p* < 0.001.

RCs: reactive carbonyl species.

## DISCUSSION

In the present study, irsogladine maleate was selected from a group of six gastric mucosal protectants following through screening methods to evaluate their suppressive activities on multiple oxidative stress-related transcription factors. Furthermore, Min mice treated with irsogladine maleate had a significantly reduced number of intestinal polyps.

Regarding reports of the tumor suppressive effects of irsogladine maleate, the suppressive effect of irsogladine maleate on *N*-methyl-*N’*-nitro-*N*-nitrosoguanidine-initiated and glyoxal-promoted gastric carcinogenesis has been demonstrated in male Wistar rats [10]. Twenty-five or 100 ppm irsogladine maleate in the diet for 25 weeks reduced the average numbers of digestive tract neoplasms up to 61% of the untreated group value. In addition, another report described the suppressive effects of irsogladine maleate on diethylnitrosamine (DEN)-initiated and phenobarbital-promoted hepatocarcinogenesis in male F344 rats [11]. Irsogladine maleate in the diet at 125 ppm for 35 weeks did not allow the development of liver neoplasms (incidence, 0/14 rats). As far as we know, this is the first report to examine the effects of irsogladine maleate on intestinal neoplastic lesions. Comparing the doses and treatment periods of our study with other reports, irsogladine maleate suppressed the development of intestinal neoplastic lesions stronger than its suppression of other organ-derived neoplastic lesions. Moreover, 50 ppm irsogladine maleate seems no more effective than 5 ppm. We assumed that 5 ppm irsogladine maleate is a sufficient dose to suppress intestinal polyp formation and reactive carbonyl species. The data suggested the effects of 5 ppm irsogladine maleate plateaus, and higher doses did not efficiently decrease polyp numbers. The data using 0.5 ppm, almost the same dose as in medical practice, is of interest to investigate in the future.

Among the six gastric mucosal protectants, irsogladine maleate was found to inhibit NF-κB transcriptional activity in Caco-2 cells. Similar results obtained in HCT-15 cells support the reliability of our results. NF-κB plays a critical role in host defense responses through the induction of several genes and is related to inflammatory and immunological responses [12, 13]. Furthermore, the dysregulation of NF-κB has been suggested to promote carcinogenesis [14–17]. Thus, the inhibition of NF-κB transcriptional activity by irsogladine maleate might be related to its anti-carcinogenic potential observed in this study. Of note, the potential inhibition of NF-κB by irsogladine maleate appears to be weaker than commercially available NF-κB inhibitors, such as SM-7368. For safety purposes in the long-term use of cancer chemopreventive agents, weak inhibitors may be advantageous. In fact, to our knowledge, no descriptions of severe side effects of irsogladine maleate treatment have been reported in the literature.

The nuclear translocation of NF-κB initiates NF-κB transcriptional activities that result in the expression of NF-κB responsive genes, such as IL-1β and IL-6 [18–20]. In the polyps, but not the non-polyp intestinal mucosa, of Min mice, we showed that the mRNA levels of IL-1β and IL-6 were decreased by irsogladine maleate treatment. These results suggest that irsogladine maleate may modify the development of intestinal polyps in Min mice partly through the NF-κB signaling pathway.

Treatment of Min mice with irsogladine maleate also lowered hepatic levels of RCs, which form endogenously by lipid peroxidation. Several RCs, including acetaldehyde, acrolein, 2-nonenal and HNE, are reported to cause damage to DNA and proteins, and this damage is associated with disease development, including chronic inflammation and carcinogenesis [21–23]. For example, lipid peroxidation-induced exocyclic DNA adduct levels are significantly increased in the affected organs of cancer-prone subjects suffering from Crohn's disease, chronic hepatitis, chronic pancreatitis and familial adenomatous polyposis [24]. Heptanone-etheno-DNA adducts, or lipid hydroperoxide-derived adducts, are significantly increased in Min mice compared with wild type C57BL/6J mice [25]. Therefore, the suppression of oxidative stress in Min mice by irsogladine maleate could be related to the reduction of intestinal tumorigenesis.

In conclusion, this study demonstrated that irsogladine maleate may suppress the development of intestinal polyps in Min mice, in part through the NF-κB signaling pathway, suggesting the utility of irsogladine maleate as a chemopreventive agent. Further *in vitro* and *in vivo* experiments and clinical trials investigating the suppression of colon carcinogenesis by irsogladine maleate are needed to provide solid evidence of the chemopreventive effects of irsogladine maleate in the colon. Clinical trials using a combination of aspirin and irsogladine are also required to obtain evidence of the safety of this combination method - the results of which may impact colorectal cancer prevention strategies.

## MATERIALS AND METHODS

### Chemicals

Dansyl hydrazine (DH), glyoxal, crotonaldehyde, 2,4-nonadienal (NDE), 2,4-decadienal (DDE), heptadecanal, hexadecanal, irsogladine maleate, octadecanal, pentadecanal, rebamipide, teprenone, tetradecanal and troxipide were purchased from Tokyo Chemical Industry (Tokyo, Japan). The NF-κB inhibitor SM-7368 was obtained from Merck KGaA (Darmstadt, Germany). p-Toluenesulfonic acid (p-TsOH) and the RCs including propanal, pentanal, butanal, 2-hexenal, hexanal, 2-heptenal, heptanal, octanal, 2-nonenal, nonanal, decanal, undecanal, dodecanal and tridecanal were obtained from Sigma-Aldrich (St. Louis, MO, USA). 4-Hydroxy-2-hexenal (HHE), 4-hydroxy-2-nonenal (HNE), 4-oxo-2-nonenal (ONE) and 4,5-epoxy-2-decenal (EDE) were purchased from the Cayman Chemical Company (Ann Arbor, MI, USA). Ecabet sodium hydrate, p-benzyloxybenzaldehyde (p-BOBA), sofalcone and other chemicals used in the current study were purchased from Wako Pure Chemical Industries (Osaka, Japan). 8-Heptadecenal (8-HpDE), 8,11-heptadecadienal (8,11-HpDDE) and 8,11,14-heptadecatrienal (8,11,14-HpDTE) were synthesized by a method previously described [26, 27]. Secosterol-A and -B were synthesized according to the procedure reported by Wentworth *et al*. [28]. Stock solutions of the RCs and an internal standard (IS) (p-BOBA, 10 mM) were prepared separately in acetonitrile and stored at −20°C prior to use.

### Cell culture

Caco-2 cells, a human colon adenocarcinoma cell line, were purchased from Sumitomo Dainippon Pharma Co., Ltd. (Osaka, Japan). HCT-15 cells were purchased from the American Type Culture Collection (Manassas, VA, USA). Caco-2 and HCT-15 cells were maintained in DMEM medium supplemented with 10% heat-inactivated fetal bovine serum (FBS; Hyclone Laboratories Inc., Logan, UT, USA) and antibiotics (100 μg/mL streptomycin and 100 U/mL penicillin) at 37°C in 5% CO_2_. The Caco-2 cells were also supplemented with MEM Non-Essential Amino Acids Solution (Nacalai Tesque, Inc., Japan).

### Animals

Male C57BL/6-*Apc^Min/^*^+^ mice (Min mice) were purchased from The Jackson Laboratory (ME, USA). The mice (n = 4 - 5) were housed in a plastic cage with sterilized softwood chips as bedding in a barrier-sustained animal room at 24 ± 2°C and 55% humidity on a 12 h light/dark cycle. Irsogladine maleate was mixed at concentrations of 5 and 50 ppm in an AIN-76A powdered basal diet (CLEA Japan, Inc., Tokyo, Japan). We calculated the dose of 5 and 50 ppm that corresponds to a human dose. As a medicine, human dosage irsogladine maleate is 4 mg/day, so we used irsogladine maleate at about 10 to a 100 times higher dose compared to the human dose.

### Animal experiment protocols

Ten male Min mice at 5 weeks of age were given 0, 5 and 50 ppm irsogladine maleate for 8 weeks. All animals in the same cage were in the same treatment group. Food and water were available *ad libitum*. The animals were observed daily for clinical signs and mortality. Body weight and food consumption were measured weekly. At the sacrifice time points, mice were anesthetized, and blood samples were collected from the abdominal vein. The levels of serum triglyceride, free fatty acid, and total cholesterol were measured as previously reported [29]. The intestinal tract was removed and separated into the small intestine, cecum and colon. The small intestine was divided into the proximal segment (4 cm in length) and the proximal (middle) and distal halves of the remainder. Polyps in the proximal segments were counted and picked under a stereoscopic microscope; the remaining intestinal mucosa (non-polyp portion) was removed by scraping, and the specimens were stored at −80°C for quantitative real-time PCR analysis. Other segments were opened longitudinally and fixed flat between sheets of filter paper in 10% buffered formalin. The numbers and sizes of polyps and their distributions in the intestine were assessed with a stereoscopic microscope. The experiments were performed according to the “Guidelines for Animal Experiments in the National Cancer Center” and were approved by the Institutional Ethics Review Committee for Animal Experimentation of the National Cancer Center.

### Immunohistochemical staining of PCNA to evaluate the proliferation of intestinal polyps in Min mice

The small intestines were fixed, embedded and sectioned as Swiss rolls for further immunohistochemical examination using the avidin–biotin complex immunoperoxidase technique after heating with 10 mM citrate buffer (pH 6.0). The primary antibody was monoclonal mouse anti-PCNA antibody (Ab) (Calbiochem, CA, USA) at a 100× dilution. As the secondary Ab, biotinylated horse anti-mouse IgG (Vector Laboratories, Burlingame, CA, USA) was used at a 200× dilution. Staining was performed using avidin–biotin reagents (Vectastain ABC reagents; Vector Laboratories), 3,3′-diaminobenzidine and hydrogen peroxide, and the sections were counterstained with hematoxylin to facilitate orientation. As a negative control, consecutive sections were immunostained without exposure to the primary Ab. The ratio of PCNA-positive cells was calculated by the formula % = the number of PCNA positive cells per polyp / the total number of cells in the polyp (100x magnification).

### Quantitative real-time polymerase chain reaction (PCR) analyses

Total RNA was isolated from intestinal polyps and non-polyp-containing intestinal mucosa using TRIzol Reagent (Invitrogen, Grand Island, NY, USA), treated with DNase (Invitrogen) and 1 μg in a final volume of 20 μL was used for cDNA synthesis using a High Capacity cDNA Reverse Transcription Kit (Applied Biosystems, Foster City, USA). Real-time PCR was conducted using a CFX96/384 (BIO RAD, Tokyo, Japan) and Fast Start Universal SYBR Green Mix (Roche Diagnostics, Mannheim, Germany) according to the manufacturers’ instructions. Primer sequences were as follows: IKBKB (5′-ATC AGG CGA CAG GTG AAC AG and 3′-GGC CAC AGC AGT TCT CGA A), IL-1β (5′-GAA ATG CCA CCT TTT GAC AGT G and 3′-TGG ATG CTC TCA TCA GGA CAG), IL-6 (5′-TGT TCT CTG GGA AAT CGT GGA and 3′-AAG TGC ATC ATC GTT GTT CAT ACA) and glyceraldehyde-3-phosphate dehydrogenase (GAPDH) (5′-TTG TCT CCT GCG ACT TCA and 3′-CAC CAC CCT GTT GCT GTA). To assess the specificity of each primer set, amplicons generated from the PCR reactions were analyzed for melting curves.

### Luciferase assays for AP-1, NF-κB, NRF2, p53 and STAT3 transcriptional activity

To measure AP-1, NF-κB, NRF2, p53 and STAT3 transcriptional activity, Caco-2 or HCT-15 colon cancer cells were seeded in 12-well plates (4.0 × 10^5^ cells/well). After a 24-h incubation, the cells were transiently transfected with 1 μg/well of the pAP1-Luc, pNF-κB-Luc, pNRF2/ARE-Luc, pP53-Luc, pSTAT3-Luc or pTA-Luc (Signosis Inc., Santa Clara, CA, USA) reporter plasmid and 100 ng/well pGL4.73 [hRluc/SV40] control plasmid (Promega, Madison, WI, USA) using Polyethylenimine Max MW 40,000 (PolyScience, Warrington, PA, USA); transfected cells were cultured for an additional 24 h, treated with the test agents (Figure 1) for 24 h and firefly and Renilla luciferase activities were determined using the Bright GLO and Renilla GLO Systems (Promega), respectively. In the case of the cytokine mixture experiment, transfected cells were cultured in the presence of 50 ng/mL TNFα (Perotec, NJ, USA), 5 ng/mL IL-1β and 50 ng/mL EGF (Miltenyi Biotec Inc., CA, USA) for 24 h and 48 h after 30 min incubation with irsogladine maleate. The basal luciferase activity of NF-κB in the control was set as 1.0. The percentage luciferase activity with each treatment was calculated from the data of triplicate wells, with values normalized by those of the Renilla luciferase activity. The data are expressed as the means ± SD (n = 4).

### Reactive carbonyl species extraction and LC/MS methodology

Details of RCs extraction and the LC/MS method were described previously [30]. Mouse livers (20 mg) were homogenized in 200 μL of sodium phosphate buffer (50 mM, pH 7.4) containing 0.5 mM EDTA and 20 μM BHT. Liver homogenates were mixed with an IS (p-BOBA) (20 pmol) and 400 μL of a chloroform/methanol (2:1, v/v) solution; the resulting mixture was vigorously agitated for 1 min then centrifuged at 15,000 rpm for 10 min and the organic phase was collected. The remaining precipitate and aqueous phases were then mixed with 400 μL of chloroform/methanol solution (2:1, v/v), and the resulting mixture was centrifuged at 15,000 rpm for 10 min to obtain the organic phase. The combined organic phases were mixed with 100 μL of acetonitrile containing 50 μg of DH and 10 μg of p-toluenesulfonic acid and incubated for 4 h at ambient temperature in the absence of light. The mixtures were then evaporated to dryness *in vacuo* to give the corresponding derivatized residues. These residues were dissolved in 200 μL of acetonitrile, and 5 μL of sample were injected onto the LC/MS system.

The RC-DH derivatives were separated on a TSK-gel Super Octyl column (2.3 μm, 100 × 2.0 mm, TOSOH, Tokyo, Japan) connected to an Agilent 1200 series HPLC system and an Agilent G6410B triple quadrupole tandem mass spectrometer with electrospray ionization (Agilent Technologies, Santa Clara, CA, USA). Mobile phase A consisted of a 0.1% (v/v) solution of formic acid in water, and mobile phase B consisted of a 0.1% (v/v) solution of formic acid in acetonitrile. The linear gradient conditions were as follows: 20% B at 0 min, 100% B at 10 min, 100% B at 20 min and 20% B at 20.01 min, followed by a 9.99 min equilibration time. The system was operated at a constant flow rate of 0.2 mL/min for all of the analyses. The capillary voltage was set to 4000 V, with a source temperature of 300°C, a drying gas flow rate of 7 L/min, a nebulizer gas pressure of 20 psi, the fragmentor at 200 V and collision energies of 13 eV (m/z 275–349), 20 eV (m/z 350–449) and 29 eV (m/z 450–949). Nitrogen was used as the collision gas. The RC-DH derivatives were detected using the selected reaction monitoring (SRM) mode. This strategy was designed to detect a specific ion product with an m/z value of 236.1 by CID. This ion product was assigned to the 5-dimethylaminonaphthalene-1-sulfonyl moiety derived from the positively ionized DH derivatives. The RCs-DH derivatives were therefore specifically and sensitively detected by monitoring their transmitting [M+H]+→236.1 transitions. A total of 675 SRM transitions were monitored for each DH-derivatized sample, with the transitions ranging from m/z 275→236.1 to 949→236.1. For each sample injection, a total of 100 channels were monitored simultaneously. One channel for each injection was reserved to monitor the transition of the IS at m/z 460→236.1. Each sample was injected seven times to complete the monitoring of the 675 SRM transitions.

### Statistical analyses

All results are expressed as the means ± SD values, with statistical analyses performed using Student's t-tests. The exceptions are the NF-κB transcriptional activity investigation in Caco-2 and HCT-15 cells, the examination of intestinal polyp formation/size distribution and serum lipid concentrations, which were analyzed by Bonferroni's test. Differences were considered to be statistically significant at *p < 0.05, **p < 0.01 and ***p < 0.001.

## SUPPLEMENTARY FIGURES



## Abbreviations

AP-1: activator protein-1
COX: cyclooxygenase
DEN: diethylnitrosamine
FBS: fetal bovine serum
GAPDH: glyceraldehyde-3-phosphate dehydrogenase
IKBKB: inhibitor of nuclear factor kappa-B kinase subunit beta
IL: interleukin
IS: internal standard
LC/MS: liquid chromatography mass spectrometry
NF-κB: nuclear factor-kappaB
NRF2: NF-E2-related factor 2
NSAID: non-steroidal anti-inflammatory drug
PCR: polymerase chain reaction
PCNA: proliferating cell nuclear antigen
RCs: reactive carbonyl species
SRM: selected reaction monitoring
STAT3: signal transducer and activator of transcription 3.
