# Supplementary material for: Irsogladine maleate, a gastric mucosal protectant, suppresses intestinal polyp development in Apc-mutant mice
Source: Oncotarget. 2016 Jan 30;7(8):8640–52. doi: 10.18632/oncotarget.7082 (PMC4890993; doi:10.18632/oncotarget.7082)
Supplement: Supplementary file 1 [file oncotarget-07-8640-s001.pdf]

SUPPLEMENTARY FIGURES

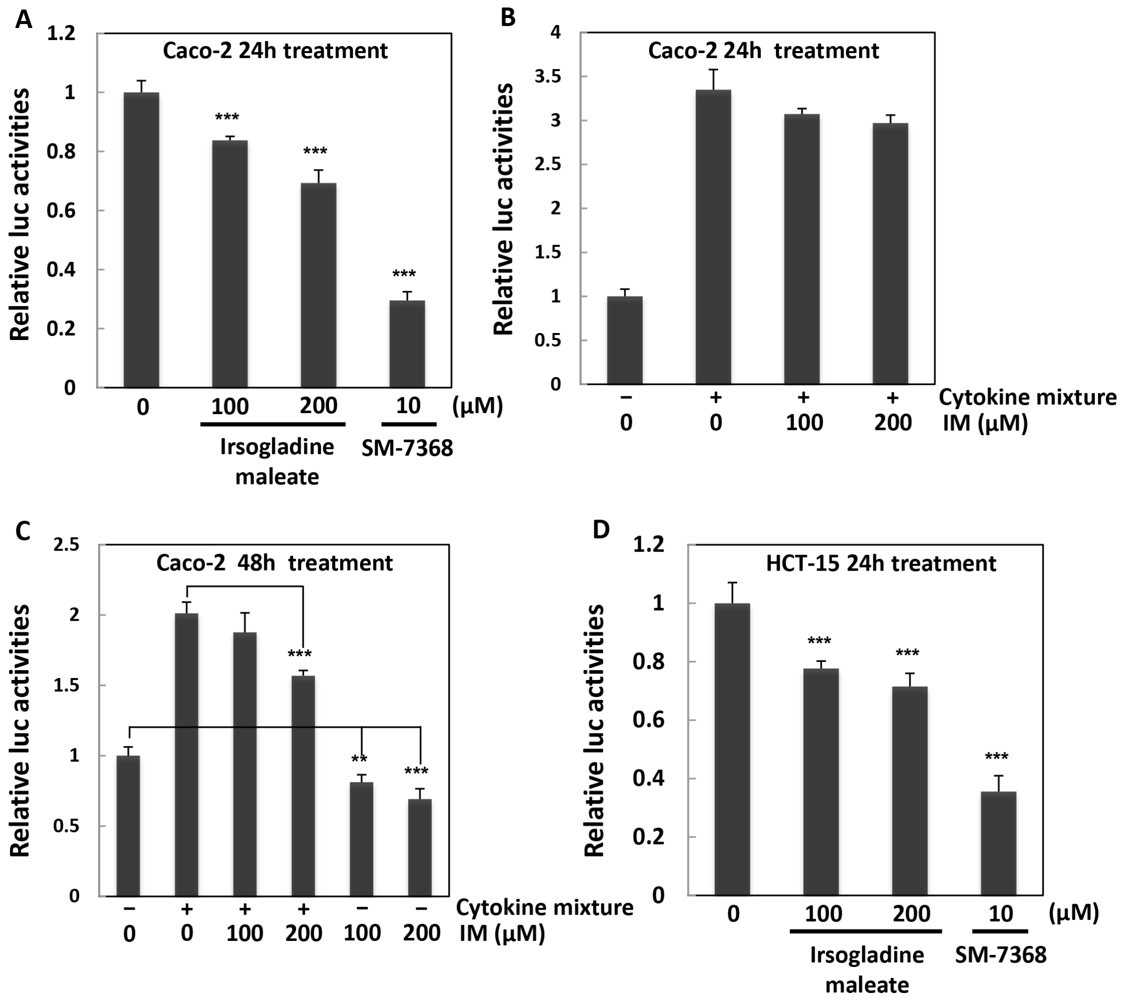

Supplementary Figure S1: Suppression of NF-κB transcriptional activation by irsogladine maleate or NF-κB inhibitors in human colon cancer cells. Caco-2 cells **A.** and HCT-15 cells **D.** were treated with irsogladine maleate or the NF-κB inhibitorSM-7368 for 24 h. Caco-2 cells were cultured in the presence of 50 ng/mL TNFα, 5 ng/mL IL-1β and 50 ng/mL EGF for 24 h **B.** and 48 h **C.** after 30 min incubation with irsogladine maleate. The basal luciferase activity of the control was set as 1.0. The data are the means ± SD (n = 4), ***$p < 0.001$, **$p < 0.01$ *vs* control. IM: irsogladine maleate.

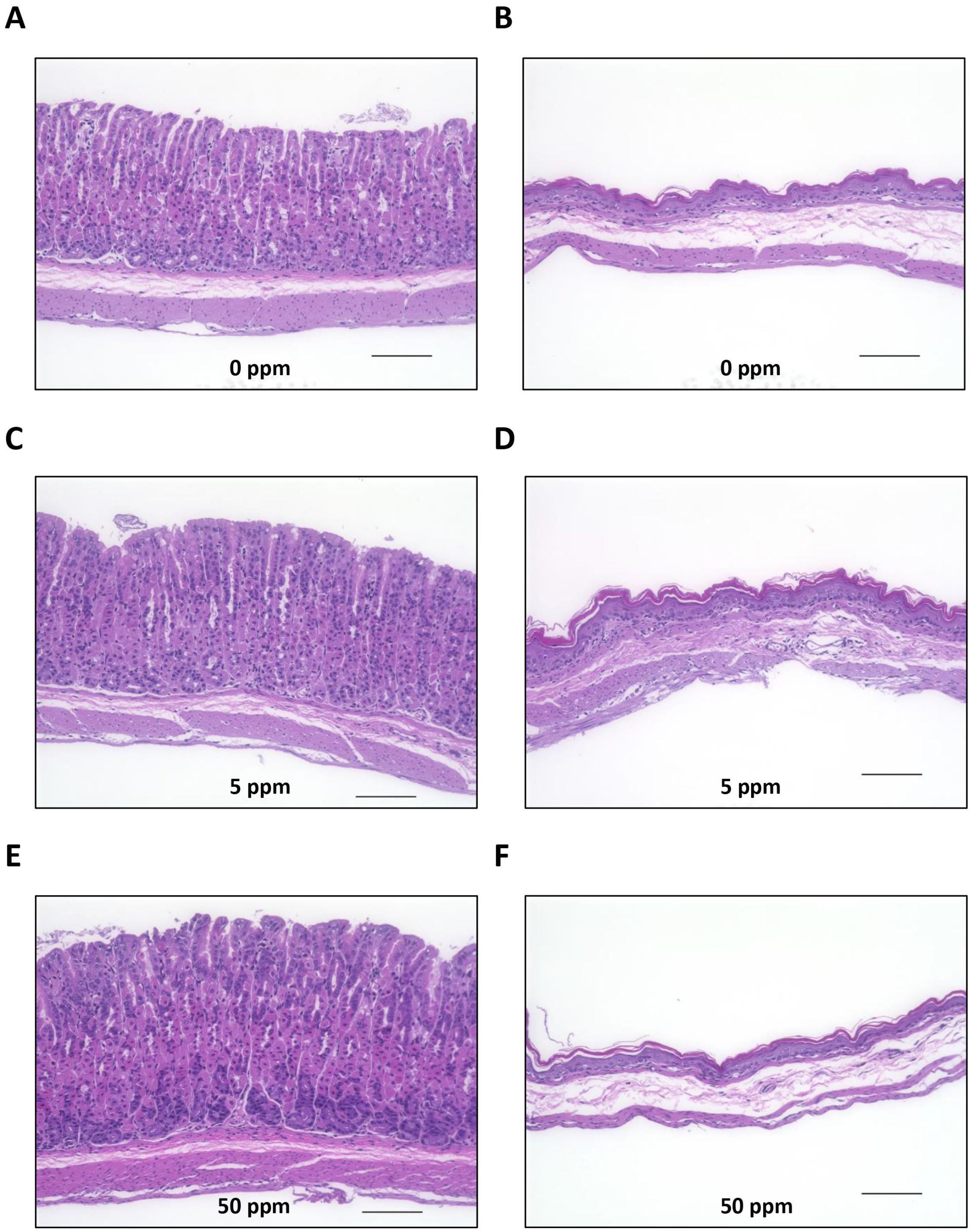

Supplementary Figure S2: Typical microscopic views of glandular stomach of Min mice with or without irsogladine maleate treatment. Photograph of representative H.E. staining of small glandular stomach **A, C, E.** and forestomach **B, D, F.** in the three groups is shown. Basal diet group (A, B); 5 ppm group (C, D); 50 ppm group, (E, F). Bar=100 µm.

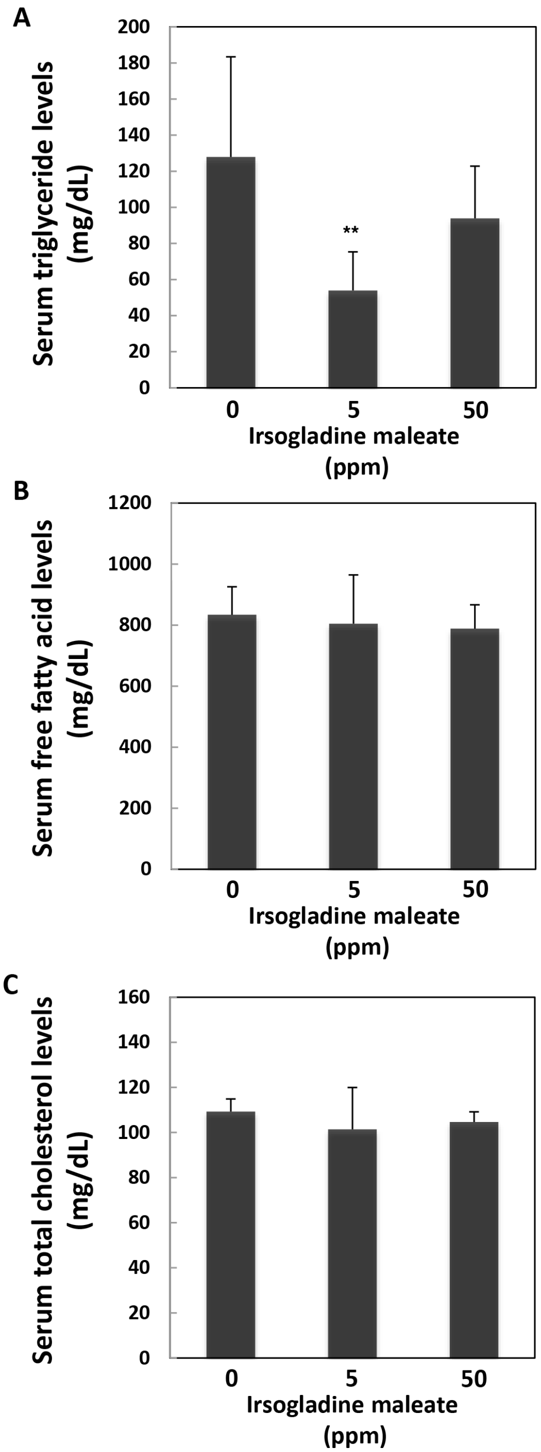

Supplementary Figure S3: Levels of serum lipids in Min mice with or without irsogladine maleate treatment. Serum triglyceride levels **A.** serum free fatty acid levels **B.** and serum total cholesterol levels **C.** are shown in 0, 5 and 50 ppm irsogladine maleate-treated groups. The data are the means ± SD (n = 8), $^{**}p < 0.01$ *vs* 0 ppm.

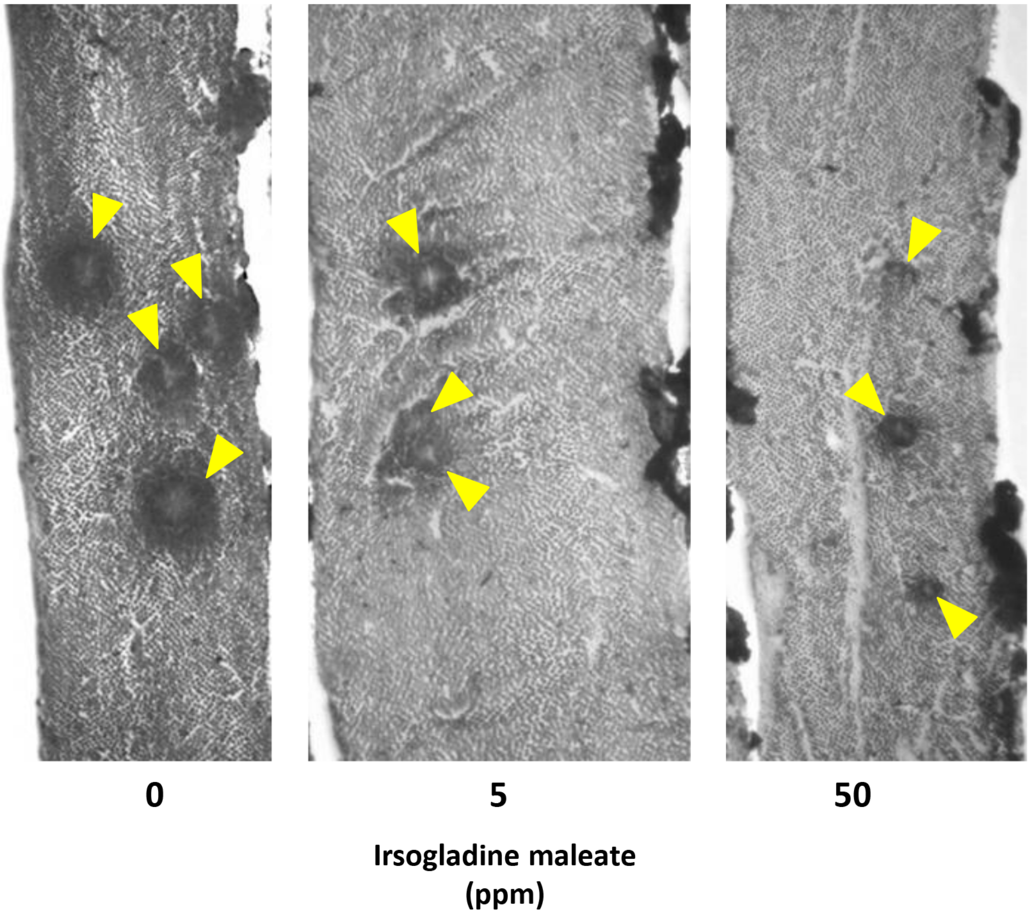

Supplementary Figure S4: Representative photos of small intestinal polyps in the three groups. The polyps in the distal parts of the small intestine were photographed under a microscope. Basal diet group, left; 5 ppm group, middle; 50 ppm group, right. Arrow head indicates polyp.

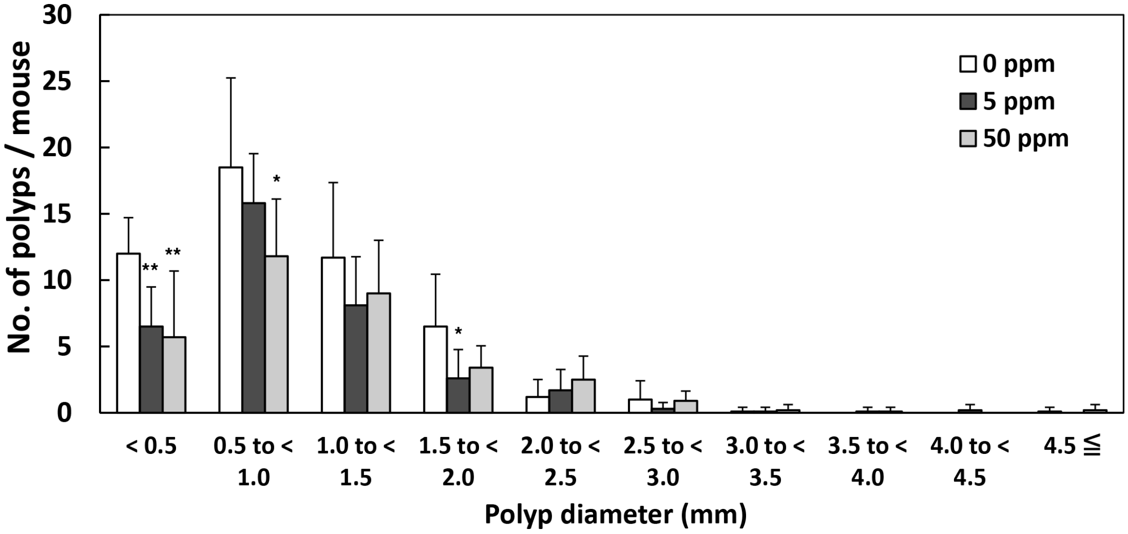

Supplementary Figure S5: Effects of irsogladine maleate on the size distribution of intestinal polyps in Min mice. Min mice were fed a basal diet (open box) or a diet containing 5 ppm (black-filled box) or 50 ppm (gray-filled box) irsogladine maleate for 8 weeks. The number of polyps per mouse in each size class is given as the mean ± SD. $^{**}p < 0.01$, $^{*}p < 0.05$ *vs* 0 ppm.

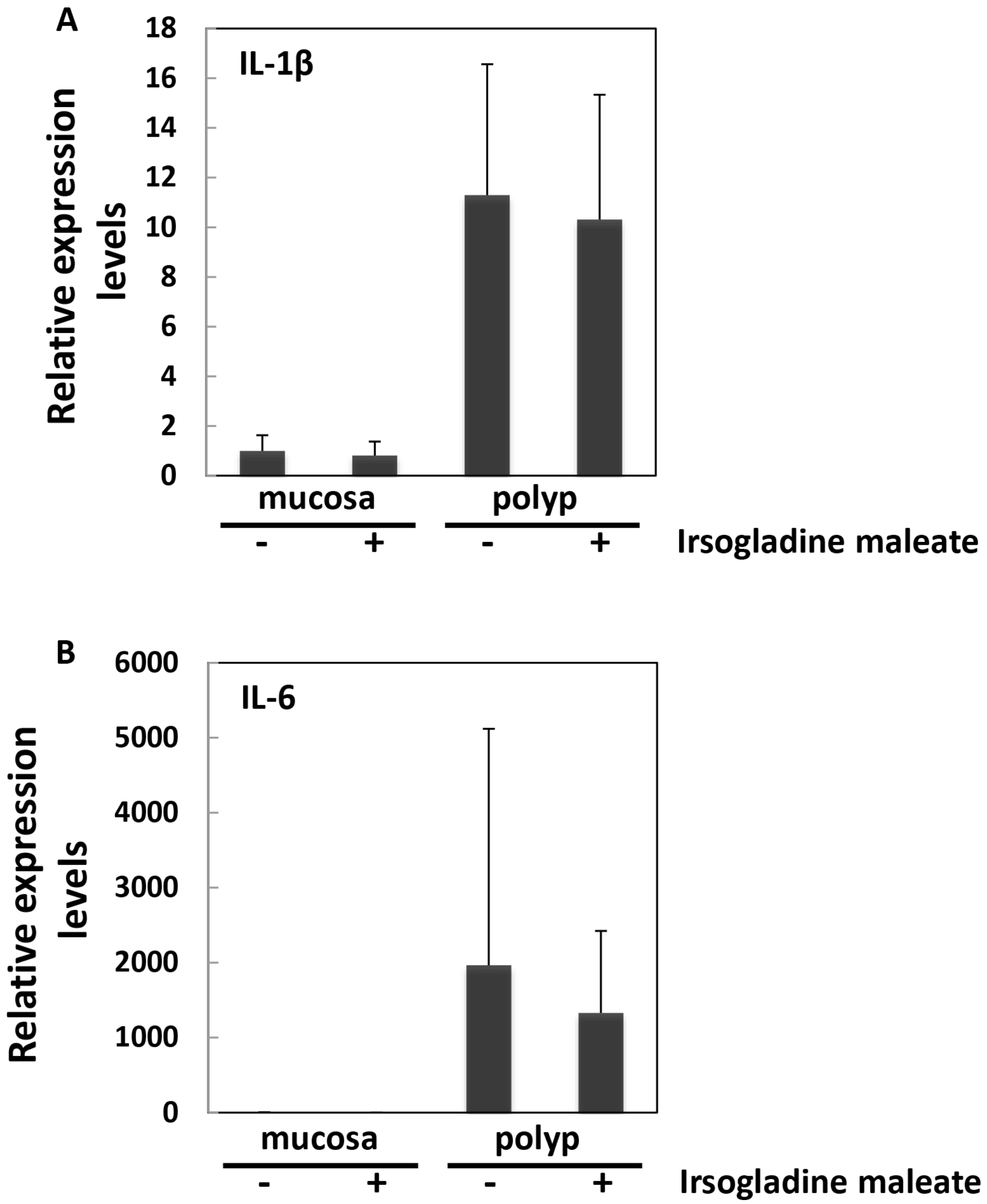

Supplementary Figure S6: Suppression of the down-stream target genes of NF-κB in non-polyp intestinal mucosa segments and/or polyp segments of Min mice with or without 50 ppm irsogladine maleate treatment. Quantitative real-time PCR analyses were performed to determine the IL-1β **A.** and IL-6 **B.** mRNA expression levels in the polyps or non-polyp intestinal mucosa of Min mice that received diets containing irsogladine maleate at doses of 50 ppm for 8 weeks. The data are normalized according to GAPDH and presented as the means ± SD.
